# Oncogenic β-catenin stimulation of cofilin 1-mediated macropinocytosis is druggable for cancer

**DOI:** 10.7150/thno.104283

**Published:** 2025-03-17

**Authors:** Baohui Zhang, Xiaochen Gai, Bufu Tang, Linlin Chen, Yuting Wu, Weiwei Deng, Jiate Liu, Lanting Hao, Fangming Liu, Hongbing Zhang

**Affiliations:** 1 Department of Physiology, School of Life Science, China Medical University, Shenyang, Liaoning, China.; 2 Department of Physiology, State Key Laboratory of Common Mechanism Research for Major Diseases, Haihe Laboratory of Cell Ecosystem, Institute of Basic Medical Sciences and School of Basic Medicine, Chinese Academy of Medical Sciences and Peking Union Medical College, Beijing, China.; 3 Department of Radiation Oncology, Zhongshan Hospital, Fudan University, Shanghai, China.; 4 School of Life Sciences, Westlake University, Hangzhou, Zhejiang, China.; 5 Department of Neurosurgery, Shengjing Hospital of China Medical University, Shenyang, Liaoning, China.; 6 Department of Surgical Oncology and General Surgery, The First Hospital of China Medical University, Shenyang, Liaoning, China.; 7 Hubei Provincial Key Laboratory of Tumor Microenvironment and Immunotherapy, College of Basic Medical Sciences, China Three Gorges University, Yichang, Hubei, China.

**Keywords:** β-catenin, liver cancer, macropinocytosis, methuosis, CFL1

## Abstract

**Rationale:** Although β-catenin is frequently activated in various cancers, no targeted therapies have been approved for clinical use.

**Methods:** High-throughput drug screening was performed to identify potential compounds against β-catenin-activated tumors. The efficacy of identified compounds was assessed in orthotopic β-catenin-driven hepatocellular carcinoma model mice.

**Results:** OSI-027 emerged as the most potent agent that selectively inhibited β-catenin-mutant cells. Mechanistically, β-catenin enhanced the transcription of Cofilin 1 (CFL1), a key stimulator of macropinocytosis, and directly interacted with CFL1 to prevent its inactivation. OSI-027 induced macropinocytosis and subsequently led to methuosis-like cell death of β-catenin-mutant cells. Moreover, both excessive macropinocytosis induced by OSI-027 and macropinocytosis inhibition via CFL1 depletion suppressed β-catenin-driven tumor growth in orthotopic hepatocellular carcinoma model mice.

**Conclusion:** Targeting macropinocytosis represents a promising therapeutic strategy for β-catenin mutant cancers.

## Introduction

β-catenin signaling pathway is frequently activated in various cancers 1-3. Physiologically, phosphorylation of serine/threonine in exon 3 leads to ubiquitination-mediated degradation of β-catenin 4. However, mutations in exon 3 protect β-catenin protein from degradation and constitutively activate β-catenin signaling cascade. Notably, hyperactive β-catenin, especially the exon 3 deleted form, has been shown to be oncogenic 5. Nuclear translocated β-catenin stimulates transcription of target genes by interacting with TCF/LEF transcription factors to promote cancer development 6. However, interventions of different stages of the β-catenin signaling pathway have yet to demonstrate clinical efficacy. Novel therapeutic strategies targeting oncogenic β-catenin remain to be explored.

Macropinocytosis is an endocytic process by which extracellular materials are internalized in eukaryotic cells 7. Genetic alterations in certain driver genes stimulate macropinocytosis which provides nutrients for cancer cell proliferation 8. Oncogenic RAS-stimulated macropinocytosis is essential for the metabolic adaptation of RAS-mutant tumor cells 9. Gain-of-function mutations in PI3K or loss-of-function mutations in PTEN promote prostate cancer cell survival and proliferation under nutrient stress by activating macropinocytosis 10. In addition, the Hippo/Yap signaling pathway transcriptionally induces macropinocytosis to sustain tumor cell growth 11. Therefore, targeting macropinocytosis holds immense potential as a therapeutic strategy for cancers harboring specific genetic alterations.

Although canonic Wnt signaling can induce macropinocytosis 12, little is known about the molecular steps mediating oncogenic β-catenin induction of macropinocytosis. Moreover, whether β-catenin mutant cancers are susceptible to macropinocytosis-targeted therapies remains to be determined. In this study, we demonstrated that β-catenin-stimulated macropinocytosis rendered β-catenin-activated cells susceptible to methuosis-like cell death. We then explored the potential of various strategies for targeting macropinocytosis in the treatment of β-catenin-activated liver cancer.

## Results

### β-catenin active cells are susceptible to OSI-027

**Figure 1:**
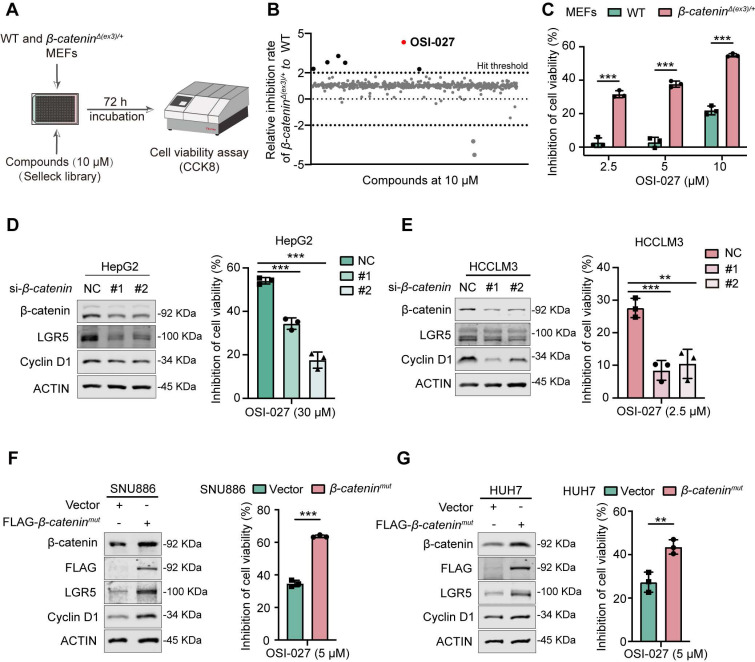
** Identification of OSI-027 as a selective inhibitor against β-catenin active cells. (A)** High-throughput drug screening strategy for identification of small molecules preferentially inhibiting the proliferation of β-catenin-activated MEFs. WT and *β-catenin^Δ(ex3)/+^* MEFs were treated with each compound (10 μM) from SELLECK Library for 72 h. Growth inhibition was determined by CCK8 assay. **(B)** Relative inhibition rate of 2148 compounds. Each dot represents one compound. Positive hits were defined as those that resulted in inhibition rate in β-catenin^Δ(ex3)/+^ MEFs being two-fold higher than in WT MEFs. OSI-027 emerged as the top 1 compound which selectively suppressed *β-catenin^Δ(ex3)/+^* cells. **(C)** Inhibition of cell viability. WT and *β-catenin^Δ(ex3)/+^* MEFs were treated with OSI-027 at different concentrations for 48 h. **(D-G)** Protein levels and inhibition of cell viability. β-catenin-mutant liver cancer cells were transfected with siRNA to knock down β-catenin. Twenty-four hours after transfection, cells were treated with DMSO or OSI-027. **(D)** HepG2, **(E)** HCCLM3. β-catenin-WT liver cancer cells were transfected with vector or *β-catenin^mut^* plasmid. Twenty-four hours after transfection, cells were treated with DMSO or OSI-027. **(F)** SNU886, **(G)** HUH7. n=3. Data were shown as mean ± SD and analysis was performed using *t test*. **p < 0.01, ***p < 0.001.

To identify potential chemicals preferentially inhibiting β-catenin-activated cells, we first established constitutively β-catenin-activated (*β-catenin^Δ(ex3)/+^*) MEFs by deleting exon 3, the most frequently altered region of *CTNNB1* (encoding β-catenin) gene in cancers 13. We then conducted a high-throughput drug screening of *β-catenin^Δ(ex3)/+^* MEFs and WT MEFs using a chemical library containing 2148 bioactive compounds (**Figure 1A**). Among these chemicals, OSI-027 emerged as the most selective compound in suppressing the viability of *β-catenin^Δ(ex3)/+^* MEFs in a dose dependent manner (**Figure 1B, C**). Because β-catenin is the most frequently altered proto-oncogene in liver cancer, we examined the susceptibility of β-catenin-activated liver cancer cells to OSI-027. Knocking down β-catenin in *CTNNB1*-mutated liver cancer cells (HepG2 and HCCLM3) abrogated the sensitivity of these cells to OSI-027 (**Figure 1D, E**). Consistently, ectopic expression of oncogenic *β-catenin^mut^* rendered β-catenin-WT liver cancer cells (SNU886 and HUH7) susceptible to OSI-027 (**Figure 1F, G**). Here, Cyclin D1 and LGR5, well-recognized genes transcriptionally regulated by β-catenin, were used as positive controls to validate β-catenin pathway activity (**Figure 1D-G**). As OSI-027 is an mTOR inhibitor 14, we assumed that mTOR activation mediated the preferential inhibition of OSI-027 on β-catenin-activated cells. However, mTOR signaling pathway was not altered by activated β-catenin (**Figure S1A**). Moreover, the classical mTOR inhibitor rapamycin has no selective inhibition on β-catenin-activated cells (**Figure S1B**). Therefore, β-catenin-activated cells are susceptible to OSI-027, likely independent of mTOR activation.

### Hyperactive β-catenin stimulation of macropinocytosis confers cells susceptible to OSI-027-triggered methuosis

**Figure 2:**
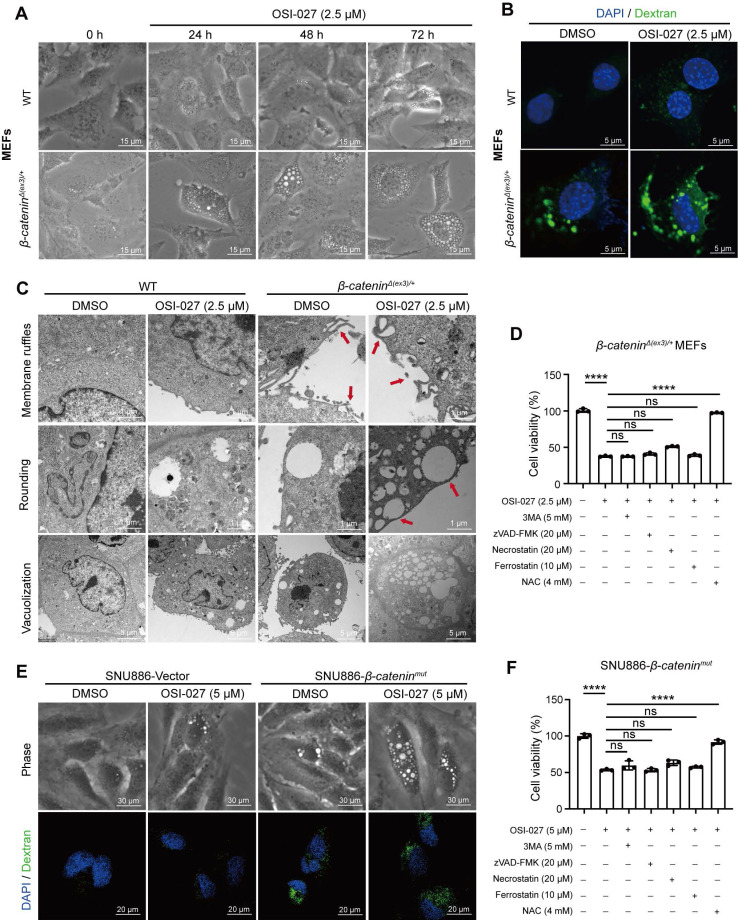
** OSI-027 induces methuosis-like cell death of β-catenin active cells. (A)** Phase-contrast images of MEFs treated with OSI-027 (2.5 μM) at different time points. Scale bars, 15 µm (20 × image).** (B)** FITC-dextran uptake of MEFs. Cells were treated with DMSO or OSI-027 (2.5 μM) for 48 h, followed by incubation with FITC-dextran (1 mg/mL) for 30 min. FITC-dextran (green), DAPI (blue). Scale bars, 5 µm (40 × image). **(C)** TEM (Transmission electron microscope) images of DMSO- and OSI-027-treated (2.5 μM, 48 h) MEFs. Red arrows indicate the formation of membrane ruffles and plasma membrane-bound vacuoles. **(D)** Inhibition rate. *β-catenin^Δ(ex3)/+^* MEFs were treated with OSI-027 in the presence or absence of specific inhibitors for autophagy (5 mM, 3MA), apoptosis (20 μM, zVAD-FMK), necroptosis (20 μM, Necrostatin), ferroptosis (10  μM, Ferrostatin) or antioxidant N-acetyl-L-cysteine (4 mM NAC). **(E)** Phase-contrast and fluorescent images of vector or *β-catenin^mut^* plasmid transfected SNU886 cells. Cells were treated with DMSO or OSI-027 (5 μM, 48 h). FITC-dextran (green), DAPI (blue). Scale bars, (upper) 30 µm (40 × image), (down) 20 µm (40 × image). **(F)** Inhibition rate. *β-catenin^mut^* SNU886 were treated with OSI-027 in the presence or absence of specific inhibitors or antioxidant N-acetyl-L-cysteine (4 mM NAC); Pictures are representative of the results from three independent replicates. Data were shown as mean ± SD and analysis was performed using *t test*. ***p < 0.001, ****p < 0.0001.

Since OSI-027 has been reported as an inducer of macropinocytosis 15, we speculated that macropinocytosis might be involved in the susceptibility of β-catenin-activated cells to OSI-027. We therefore evaluated macropinocytosis of WT and β-catenin-activated cells with or without OSI-027 treatment. Under phase contrast microscope, numerous vacuoles were present in *β-catenin^Δ(ex3)/+^* cells, whereas few vacuoles were observed in WT cells (**Figure 2A**). Moreover, OSI-027 induced catastrophic vacuolization in *β-catenin^Δ(ex3)/+^* cells but not in WT cells (**Figure 2A, Figure S2A**). As extracellular tetramethylrhodamine-labelled high-molecular-mass dextran (TMR-dextran) is a macropinosome tracer 16, we examined the uptake of TMR-dextran by these cells. *β-catenin^Δ(ex3)/+^* cells other than WT cells absorbed TMR-dextran. OSI-027 promoted the uptake of TMR-dextran by *β-catenin^Δ(ex3)/+^* cells, which was abrogated by Bafilomycin A1 (BafA1), a vacuolar-type H+-ATPase inhibitor inhibiting vacuolization of late endosomes 17 (**Figure 2B, Figure S2B**). Moreover, *β-catenin^Δ(ex3)/+^* MEFs were positive for early endosome marker EEA1 and late endosomal marker LAMP1 which were amplified by OSI-027 (**Figure S2C**). Neither lysoTracker nor mitoTracker overlapped with the vacuoles (**Figure S2D, E**), indicating that the vacuoles were not derived from lysosomes or mitochondria. Using living cell imaging and transmission electron microscope, we found that OSI-027 caused methuosis-like cell death characterized by catastrophic vacuolization in *β-catenin^Δ(ex3)/+^* MEFs (**Figure 2C**). Notably, inhibition of cell viability by OSI-027 could not be rescued by inhibitors of apoptosis, autophagy, necrosis or ferroptosis (**Figure 2D-F**). Antioxidant N-Acetylcysteine (NAC) has been reported to diminish OSI-027-induced vacuole formation 18. NAC treatment reversed the suppression of cell viability by OSI-027, indicating that OSI-027 inhibited β-catenin-activated cells resulting from massive vacuolization (**Figure 2D-F**). Furthermore, activated β-catenin not only stimulated macropinocytosis in human liver cancer cells but also sensitized these cells to OSI-027 induced methuosis-like cell death (**Figure 2E**). To further validate the susceptibleness of β-catenin-activated cells to methuosis-like cell death, we treated cells with another macropinocytosis booster MOMIPP 17. Compared to WT cells, MOMIPP selectively suppressed β-catenin-activated cells (**Figure S3A-C**). MOMIPP also triggered catastrophic vacuolization and TMR-dextran uptake in cells with activated β-catenin (**Figure S3D, E**). Therefore, β-catenin activation stimulates macropinocytosis which confers cell susceptible to OSI-027/MOMIPP-induced methuosis-like cell death.

### β-catenin transcriptionally activates CFL1

**Figure 3:**
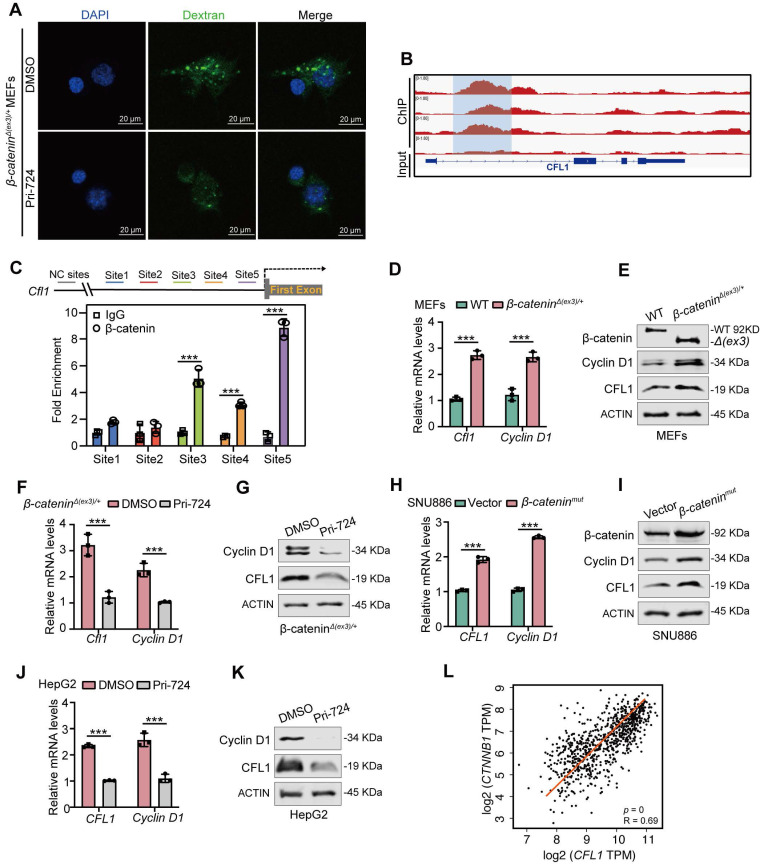
** β-catenin transactivates CFL1 expression. (A)** FITC-dextran uptake of DMSO- or Pri-724 (20 μM, 24 h)-treated *β-catenin^Δ(ex3)/+^* MEFs. FITC-dextran (green), DAPI (blue). Scale bars, 20 µm (40 × image).** (B)** β-catenin ChIP-seq profiles at *Cfl1* loci.** (C)** β-catenin ChIP-qPCR on *Cfl1* promoter was performed with *β-catenin^Δ(ex3)/+^* MEFs. Primers around peak sites from β-catenin ChIP-seq signals were used. **(D, E)** *Cfl1* mRNA levels **(D)** and protein levels **(E)** in WT and *β-catenin^Δ(ex3)/+^* MEFs. β-catenin^Δ(ex3)/+^ represents a deletion of exon 3, which removes GSK-3β phosphorylation sites required for β-catenin degradation and results in a smaller protein product. **(F, G)** *Cfl1* mRNA levels **(F)** and protein levels **(G)** in *β-catenin^Δ(ex3)/+^* MEFs treated with DMSO or Pri-724 (20 μΜ) for 48 h. **(H, I)** *CFL1* mRNA levels **(H)** and protein levels **(I)** in SNU886 transfected with vector or *β-catenin^mut^* plasmid. **(J, K)** *CFL1* mRNA levels** (J)** and protein levels** (K)** in HepG2 treated with DMSO or Pri-724 (20 μM) for 48 h. **(L)** Correlation between *CTNNB1* and *CFL1* expression in human HCC, COAD and UCEC. The original data were from TCGA database. ChIP-qPCR and qPCR, n=3. ***p < 0.001. Data were shown as mean ± SD. Analysis was performed using *t test*.

Since β-catenin functions as a transcriptional co-activator 19, it may transcriptionally regulate macropinocytosis-associated genes. We found that β-catenin inhibitor Pri-724 suppressed β-catenin-induced macropinocytosis in both MEFs and liver cancer cells (**Figure 3A, Figure S4A**). ChIP-seq analysis revealed the enrichment of β-catenin within the promoter region of CFL1 (**Figure 3B**), a known positive regulator of macropinocytosis 20. ChIP-qPCR was conducted to validate the binding of β-catenin to the *CFL1* promoter region (**Figure 3C**). Both mRNA and protein levels of CFL1 were significantly higher in *β-catenin^Δ(ex3)/+^* MEFs compared to WT ones (**Figure 3D, E**). Consistently, Pri-724 reduced CFL1 expression in *β-catenin^Δ(ex3)/+^* MEFs (**Figure 3F, G**). Moreover, CFL1 expression was augmented by activated β-catenin (**Figure 3H, I, Figure S4B, C**) but was reduced by Pri-724 in human liver cancer cells (**Figure 3J, K, Figure S4D, E**). Notably, *CFL1* expression is positively correlated with *CTNNB1* expression in human tumor samples (HCC, COAD, and UCEC) from the TCGA database (**Figure 3L**). Taken together, these findings indicate that β-catenin exerts transcriptional activation of CFL1.

### β-catenin interacts with CFL1 and protects CFL1 from phosphorylation

**Figure 4:**
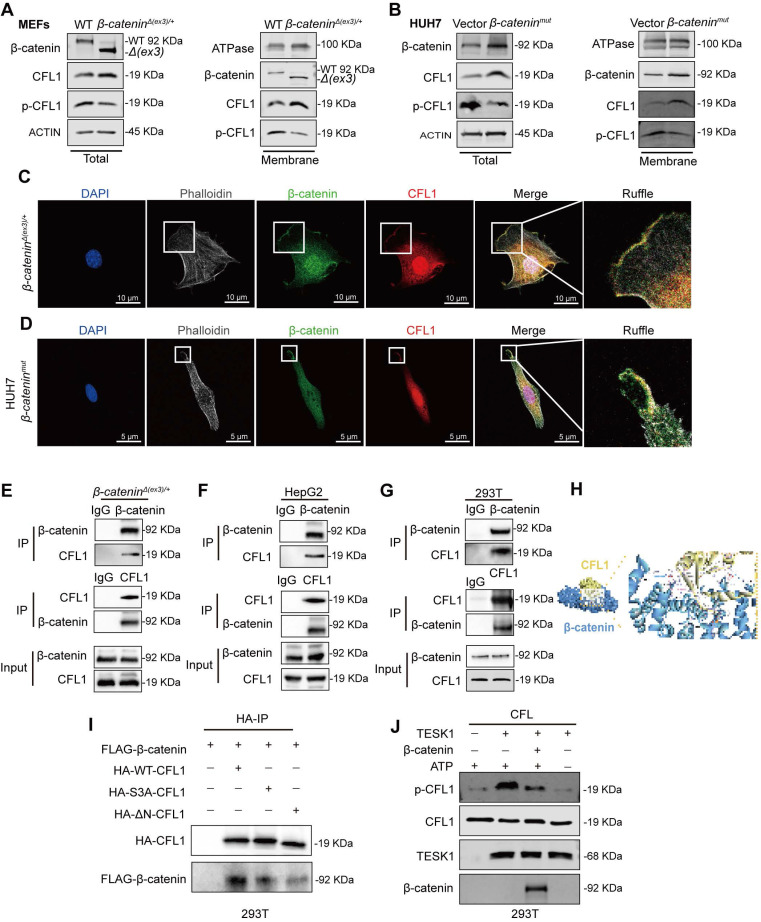
** Interaction of β-catenin with CFL1 prevents CFL1 phosphorylation. (A, B)** CFL1 and phospho-CFL1 levels in whole cell or cell membrane were examined by immunoblotting. **(A)** MEFs. **(B)** HUH7 cells were transfected with vector or *β-catenin^mut^* plasmid. (Total, left; membrane, right). **(C-D)** Immunofluorescence staining of β-catenin (green) and CFL1 (red) in *β-catenin^Δ(ex3)/+^* MEFs and *β-catenin^mut^* HUH7 cells. DAPI staining represents nucleus (blue); Phalloidin staining represents F-Actin (grey). Scale bars, 5/10 µm (40 × image). **(E-F)** Interaction between endogenous β-catenin and CFL1 in *β-catenin^Δ(ex3)/+^* MEFs **(E)** and HepG2 cells **(F)** was determined by Co-IP assays. **(G)** Interaction between exogenously expressed β-catenin and CFL1 in HEK293T cells was assessed by Co-IP assays. **(H)** Molecular docking of the interaction between β-catenin and CFL1.** (I)** Co-IP analysis of the interaction between β-catenin and WT or mutant CFL1. WT or mutant HA-CFL1 was co-expressed with FLAG-β-catenin in HEK293T cells. Immunoprecipitation by anti-FLAG antibody was performed. **(J)** *In vitro* kinase assays were performed with CFL1 and TESK1 in the presence or absence of β-catenin. CFL1 Ser3 phosphorylation was detected by immunoblotting.

CFL1 activity is regulated by Ser3 phosphorylation at its N-terminal domain 21. Testicular protein kinases (TESKs) phosphorylate Ser3 to inactivate CFL1 22. Phosphoproteome analysis of WT and *β-catenin^Δ(ex3)/+^* MEFs revealed reduced Ser3 phosphorylation of CFL1 in β-catenin mutant MEFs (**Figure S5A**). Immunoblotting confirmed that β-catenin suppressed Ser3 phosphorylation of CFL1 in both MEFs and human liver cancer cells (**Figure 4A, B, Figure S6A**). Given that both β-catenin and CFL1 have cell membrane localization in regulating actin cytoskeleton, we examined their presence on the cell membrane. In β-catenin-activated cells, β-catenin and CFL1 were increased, while p-CFL1 was decreased on the cell membrane (**Figure 4A, B, Figure S6A**). Immunofluorescence staining indicated that β-catenin colocalized with CFL1 on cell membrane ruffles (**Figure 4C, D**). Hence, we postulated that the membrane-localized β-catenin might interact with CFL1 in regulating macropinocytosis. Co-IP assay revealed the interaction between CFL1 and β-catenin (**Figure 4E-G**). Molecular docking analysis suggested that the N-terminus of CFL1 interacts with β-catenin (**Figure 4H**). β-catenin coimmunoprecipitated with exogenous wildtype, but not N-termina-deleted CFL1 (**Figure 4I**). Based on these findings, we raised the question of whether the interaction between β-catenin and CFL1 could potentially prevent the phosphorylation of CFL1. An *in vitro* kinase assay showed that β-catenin blocked the Ser3 phosphorylation of CFL1 by TESK1, a CFL1 kinase (**Figure 4J**). We concluded that β-catenin interacts with CFL1 on membrane ruffles and consequently prevents CFL1 from phosphorylation.

### CFL1 is required for β-catenin activation-stimulated macropinocytosis and tumor progression

**Figure 5:**
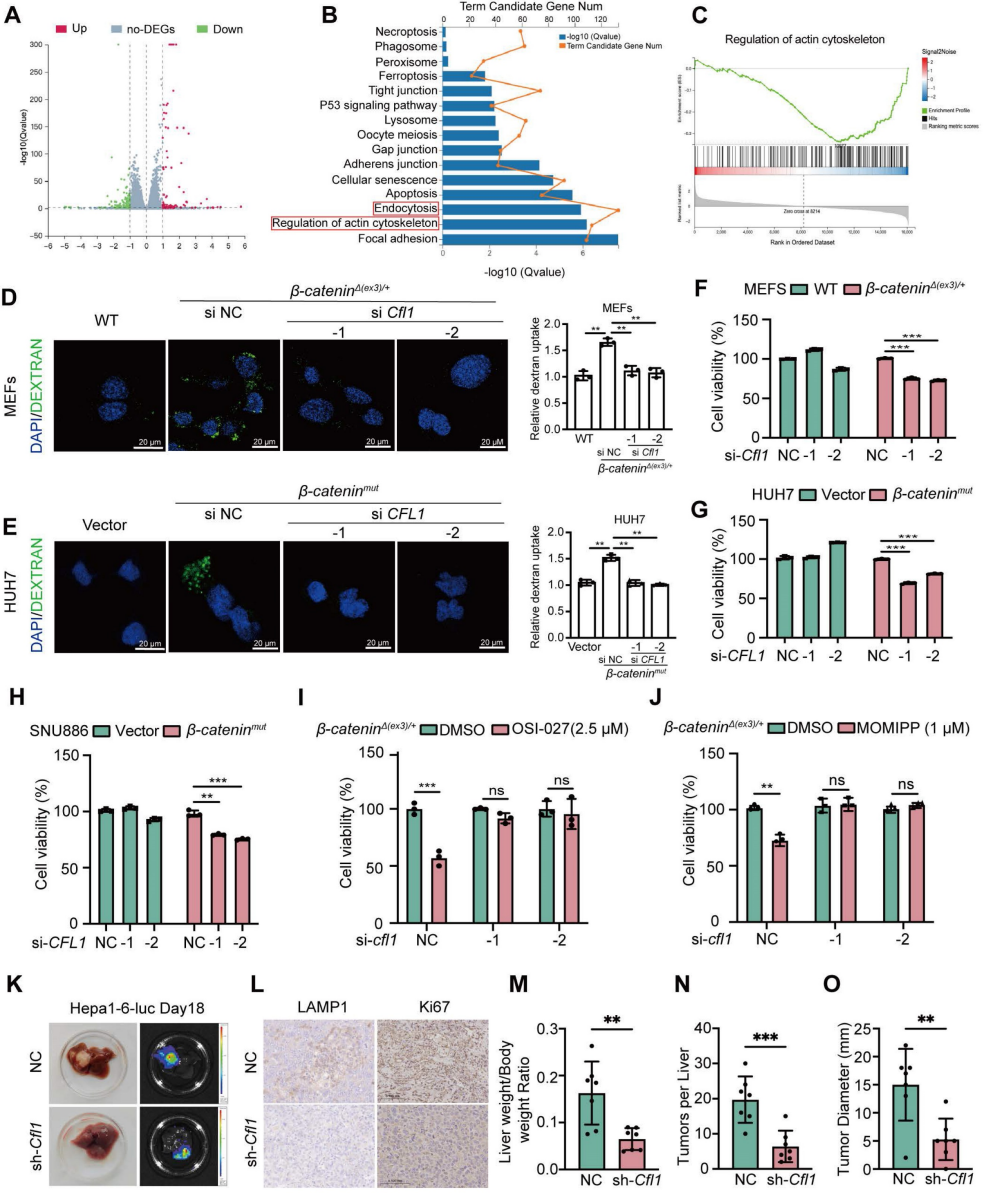
** CFL1 is required for β-catenin activation-stimulated macropinocytosis, cell proliferation and tumor formation. (A-C)** RNA-seq analysis of *β-catenin^Δ(ex3)/+^* MEFs transfected with control or *Cfl1* siRNA. ***(A)*** The volcano plot of differentially expressed genes (DEGs). Red and green indicated upregulated and downregulated genes, respectively. **(B)** KEGG functional analysis showed enriched signaling pathways associated with DEGs. **(C)** Gene Set Enrichment Analysis indicated a significant change in regulation of actin cytoskeleton signaling pathway in* β-catenin^Δ(ex3)/+^* MEFs after CFL1 knockdown. **(D, E)** FITC-dextran intake.** (D)** WT MEFs, *β-catenin^Δ(ex3)/+^* MEFs treated with control or *Cfl1* siRNA. **(E)** Vector or *β-catenin^mut^* plasmid transfected HUH7 cells were treated with negative control or *CFL1* siRNA. FITC-dextran (green), DAPI (blue). Scale bars, 20 µm (40 × image). **(F-G)** Cell viability. MEFs were transfected with negative control or *Cfl1* siRNA for 48 h. **(H)** Vector plasmid- or β-catenin^mut^ plasmid-transfected SNU886 cells were treated with control or CFL1 siRNA. **(I-J)** 48 h after transfection of control or Cfl1 siRNA, *β-catenin ^Δ(ex3)/+^* MEFs were treated with OSI-027 (2.5 μM, 48 h) **(I)** or MOMIPP (1 μM, 48 h) **(J)***.* Data were shown as mean ± SD and analysis was performed using t test. **(K)** Representative photographs and bioluminescent imagines of livers from orthotopic HCC mice planted with Hepa1-6 cells. **(L)** Representative LAMP1 and Ki67 stainings of tumors.** (M)** Ratio of liver weight to body weight.** (N)** Tumor number. **(O)** Maximum liver tumor diameter, Scale bars, 50 µm (40 × image). Data were shown as mean ± SD and analysis was performed using t test. **p < 0.01, ***p < 0.001. NC: negative control.

Given that β-catenin activates CFL1 both transcriptionally and physically, we examined the role of CFL1 in β-catenin-induced macropinocytosis and cell proliferation. RNA sequencing was performed on *β-catenin^Δ(ex3)/+^* MEFs with CFL1 knockdown. Differentially expressed genes (DEGs) were enriched in macropinocytosis-related pathways, including endocytosis and regulation of actin cytoskeleton (**Figure 5A-C**). Microscopically, depletion of CFL1 hindered the macropinocytosis provoked by active β-catenin in MEFs and liver cancer cells (**Figure 5D, E, Figure S7D**). In both MEFs and liver cancer cells, oncogenic β-catenin-promoted cell proliferation was also abolished by CFL1 depletion (**Figure 5F, G, Figure S7E**). Knocking down CFL1 also reduced the sensitivity of *β-catenin^Δ(ex3)/+^* MEFs to OSI-027 (**Figure S7F**) and another macropinocytosis inducer MOMIPP (**Figure S7G**). To assess the therapeutic potential of CFL1 inhibition in β-catenin-driven tumorigenesis, we established orthotopic liver cancer transplantation model utilizing Hepa1-6 cells with *Ctnnb1* exon 3 deletion. Depletion of CFL1 not only suppressed tumorigenicity of Hepa1-6 but also reduced LAMP1 and Ki67 expression in tumor regions (**Figure 5H-L**). Thus, CFL1 is essential for β-catenin activation-mediated macropinocytosis, cell proliferation, and tumorigenesis.

To assess the clinical relevance of CFL1 expression, we analyzed the ICGC and TCGA-LIHC databases. CFL1 levels were elevated in tumor tissues compared to adjacent normal tissues in HCC patients (**Figure S8A, B**). CFL1 expression was higher in patients with advanced tumor, node and metastasis (TNM) staging (**Figure S8C**). Increased CFL1 expression was also associated with shorter survival times in these patients (**Figure S8D, E**). Multivariate Cox regression analysis identified age (*p*<0.001), TNM stage (*p*<0.001), and CFL1 score (*p*<0.001) as independent prognostic indicators for HCC patients (**Figure S8F, G**). In addition, a nomogram was built to predict overall survival probability of HCC patients to evaluate the predictive power of CFL1 (**Figure S8H**). And the calibration curves of this nomogram further demonstrated optimal consistency of the actual likelihood with the nomogram-forecasted likelihoods (**Figure S8I-K**).

### Induction of methuosis abrogates oncogenic β-catenin-mediated tumorigenesis

**Figure 6:**
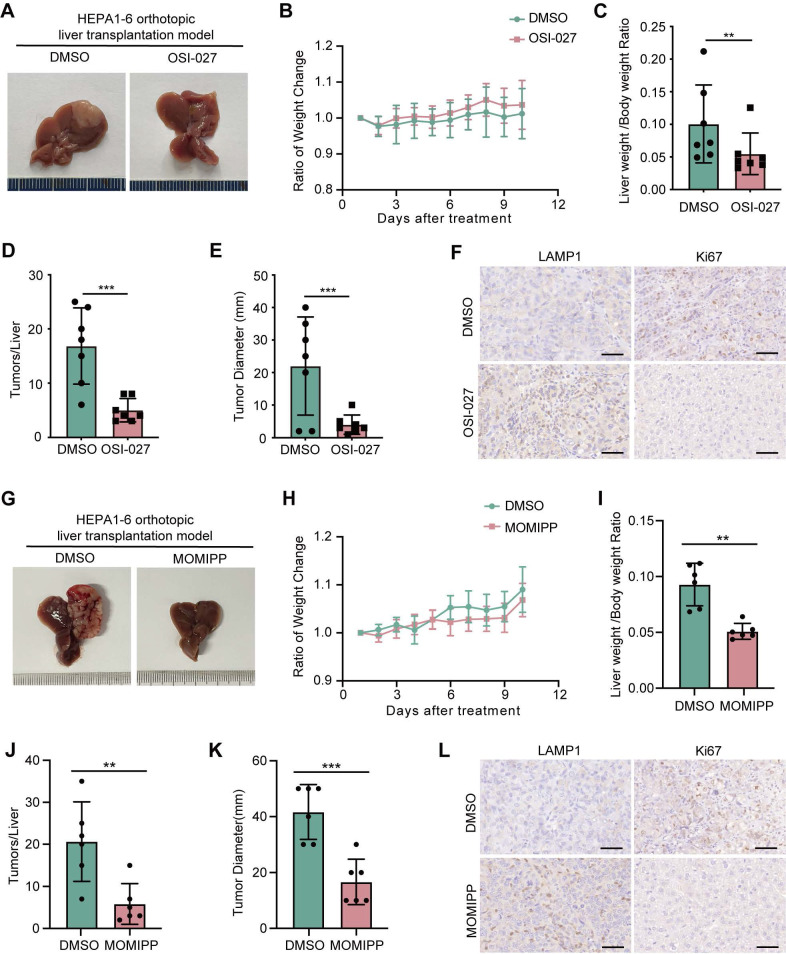
** OSI-027 and MOMIPP inhibit the growth of orthotopic liver cancer in mice. (A-F)** Orthotopic liver cancer of C57BL6 female mice intrahepatically injected with Hepa1-6 cells. Mice (n=7 each group) were administered intraperitoneally with PBS or OSI-027 (15 mg/kg, 5 day a week). **(A)** Representative morphological images of tumor nodules in liver, **(B)** Ratio of body weight change, **(C)** Ratio of liver weight to body weight, **(D)** Tumor number,** (E)** Maximum liver tumor diameter, **(F)** Representative LAMP1 and Ki67 staining of tumors, Scale bars, 50 µm (40 × image). **(G-L)** Orthotopic liver cancer of C57BL6 mice intrahepatically injected with Hepa1-6 cells. Mice (n=6 each group) were administered intraperitoneally with PBS or MOMIPP (40 mg/kg, 5 day a week). **(G)** Representative morphological images of tumor nodules in liver, **(H)** Ratio of body weight change, **(I)** Ratio of liver weight to body weight, **(J)** Tumor number, **(K)** Maximum liver tumor diameter, **(L)** Representative LAMP1 and Ki67 staining of tumors, Scale bars, 50 µm (40 × image). Data were shown as mean ± SD and analysis was performed using *t test*. **p < 0.01, ***p < 0.001.

To explore the therapeutic implications of targeting macropinocytosis in β-catenin-activated tumors, we examined the efficacy of OSI-027 or MOMIPP in suppression of tumorigenesis driven by oncogenic β-catenin in mice. Both OSI-027 and MOMIPP blocked tumor development of β-catenin-activated liver cancer without significant influence on body weight (**Figure 6A-E, G-K**). Macropinocytosis inducers also reduced LAMP1 expression in tumor regions (**Figure 6F-L**). Moreover, OSI-027 suppressed tumorigenicity of *β-catenin^Δ(ex3)/+^* MEFs without affecting body weight in nude mice (**Figure S9A-D**). Besides, there were no differences of diverse programmed cell death pathways between DMSO and OSI-027 treatment groups (**Figure S9E**). Serum levels of AST, ALT and BUN did not differ significantly between the groups, suggesting that OSI-027 did not exert observable hepatic toxicity (**Figure S9F**). Therefore, induction of massive macropinocytosis represents a therapeutic strategy for β-catenin mutant liver cancer.

## Discussion

Gain-of-function alterations of β-catenin are common in liver 23. In this study, we found that oncogenic β-catenin stimulates macropinocytosis to maintain cell proliferation and tumorigenesis. Mechanistically, β-catenin not only transcriptionally enhanced CFL1 expression but also interacted with CFL1 to protect CFL1 from phosphorylation. Exacerbating macropinocytosis by OSI-027 or MOMIPP triggered methuosis-like cell death in β-catenin-activated cells.

Despite the prevalent occurrence of active β-catenin mutations in a range of human cancers, no targeted therapy has been approved for β-catenin-activated tumors 24. Through screening 2148 bioactive compounds, we identified that OSI-027 preferentially reduced the viability of β-catenin-activated cells. We validated the selectivity of OSI-027 in inhibiting the proliferation of β-catenin hyperactive liver cancer cells. Even though OSI-027 is an mTOR inhibitor 25, we did not observe augmented mTOR signaling in β-catenin-activated cells compared to WT cells. Moreover, the classical mTOR inhibitor rapamycin did not exhibit selective suppression on β-catenin-activated cell viability. We therefore speculated that other molecular mechanisms may be responsible for the susceptibility of β-catenin-activated cells to OSI-027.

Because OSI-027 has been previously reported to induce macropinocytosis 26, we evaluated the macropinocytosis of WT and β-catenin-activated cells with or without OSI-027 treatment. β-catenin-activated cells exhibited boosted macropinocytosis while almost no macropinosomes were observed in WT cells. Further stimulating macropinocytosis by OSI-027 triggered methuosis-like cell death in β-catenin-activated cells but not in WT cells. These findings were also replicable in liver cancer cells. It has been demonstrated that further exacerbating macropinocytosis by chemicals like MOMIPP selectively triggers methuosis in cancer cells with high macropinocytic activity 27. To further validate our observations, we treated cells with another macropinocytosis inducer MOMIPP. Consistently, MOMIPP preferentially reduced the viability of β-catenin-activated cells by inducing methuosis-like cell death. These data indicated that high macropinocytosis stimulated by oncogenic β-catenin sensitizes cells to OSI-027/MOMIPP-triggered methuosis-like cell death.

Canonical Wnt signaling has been revealed to trigger the intake of extracellular fluid by macropinocytosis in supporting cancer cell growth 28. However, the molecular mechanisms underlying oncogenic β-catenin stimulation of macropinocytosis remain largely unknown, and targeting macropinocytosis in treatment of β-catenin-activated cancers needs to be determined. By analyzing β-catenin ChIP-seq data, we found that β-catenin was localized to the promoter region of CFL1. Further investigation suggested that β-catenin promoted CFL1 transcription. CFL1 binds and severs actin filaments to produce free actin spine-terminus, which is essential for phagocyte ruffling of macropinocytosis. N-terminal Ser3 is the only recognized phosphorylation site of CFL1 29. TESK1 phosphorylates Ser3 to inactivate CFL1 30. We found that β-catenin interacted with CFL1 on the cell membrane ruffles and protected CFL1 from phosphorylation by TESK1. Moreover, we demonstrated that CFL1-mediated macropinocytosis is required for β-catenin-driven cell proliferation and tumor formation. Therefore, β-catenin both transcriptionally and physically activates CFL1 to stimulate macropinocytosis and support tumor cell growth.

Multiple chemical and genetic approaches have been reported to block the viability of cancer cells with high macropinocytic activity by exacerbating macropinocytosis and stimulating methuosis 31. For example, indole-based chalcones, such as MIPP and MOMIPP, suppressed colorectal cancer cell viability by inducing methuosis. However, little is determined about the efficacy of triggering methuosis in preclinical tumor models. In this study, pharmaceutical induction of methuosis by OSI-027 or MOMIPP blocked oncogenic β-catenin-mediated tumor development.

In summary, oncogenic β-catenin stimulates macropinocytosis by upregulating CFL1 expression and suppressing CFL1 phosphorylation. β-catenin-activated cells are vulnerable to OSI-027/MOMIPP triggered methuosis. Pharmacological induction of methuosis abolishes β-catenin mutant tumor development. Thus, aggravating macropinocytosis is a promising therapeutic strategy in treatment of β-catenin-activated tumors.

## Materials and Methods

### High-throughput drug screening

The 2148-compound library was purchased from Selleck Chemicals (stock concentration: 10 mM). WT or *β-catenin^Δ(ex3)/+^* MEFs were seeded in 384-well plates (#3712, CORNING) at a density of 500 cells per well and treated with a concentration of 10 μM of each compound or DMSO using a semiautomated platform. The cells were incubated for 72 h at 37°C. Cell viability was assessed by adding CCK8 reagent ((#40203ES60, Yeasen Biotech, China) to the assay plate (3 µL per well) and measuring absorbance at 450 nm of the colored formazan product. Inhibition rate was calculated for each drug treatment using the formula as follows: Inhibition rate (%) = (OD_control group_ - OD_treated group_)/ (OD_control group_ - OD_blank group_)× 100%. The high-throughput screening was repeated 3 times. Scatterplot analysis of the average inhibition ratio of *β-catenin^Δ(ex3)/+^* relative to WT MEFs was performed to identify compounds that preferentially inhibited *β-catenin^Δ(ex3)/+^* MEFs. We selected the hit compounds based on inhibition rate in *β-catenin^Δ(ex3)/+^* MEFs being two-fold higher than in WT MEFs.

### Cell culture

WT and *β-catenin^Δ(ex3)/+^* MEFs have been described previously 32. Hepatoma cell lines HepG2, HUH7, HCCLM3 and Hepa1-6 were purchased from China Infrastructure of Cell Line Resource (Beijing, China); SNU886 cells were obtained from Cobioer Biosciences (Nanjing, China). HEK-293T were purchased from Abclonal (Wuhan, China). HepG2, HUH7, HCCLM3, Hepa1-6 and HEK-293T cells (Table S1 33) were grown in DMEM (Bological Industries, Israel) containing 10% fetal bovine serum (FBS, GIBCO, USA) and 1% penicillin/ streptomycin (P/S, Servicebio, China). SNU886 cells were maintained in RPMI-1640 (Bological Industries, Israel) medium supplemented with 10% FBS and 1% P/S. All cell lines were maintained at 37 °C incubator in an atmosphere of 5% CO_2_.

### Uptake of fluid-phase fluorescent Tracer

Cells seeded on 6 well plates were washed three times with pre-cooled PBS. FITC-dextran was diluted in preheated serum-free medium (1 mg/mL). 1 mL dextran was added in solution to each well for 30 min in the incubator. Each cover slide was washed with PBS for three times and fixed with 4% paraformaldehyde at room temperature for 15 min. The slides were mounted with DAPI after three times washes with PBS. Images were captured using Leica confocal microscope according to the FITC-dextran channel (488 nm). Five random field-of-view images were selected for each group of samples.

### *In vitro* kinase activity assay

2 μg bacterially expressed and purified TESK1 kinase were incubated with 2 μg immunoprecipitated CFL1 as substrates in the presence or absence of purified β-catenin. The reactions were started by adding 1mM ATP (#9804, Cell Signaling Technology) in 10 × kinase buffer (#9802, Cell Signaling Technology) for 30 min at 30°C. Kinase activity was detected by Western blotting with anti-pCFL1-S3 antibody (#AP0178, Abclonal).

### Plasma membrane protein isolation

Membrane fraction was isolated using the Minute Plasma Membrane Protein Isolation Kit (Invent Biotechnologies, USA) according to the manufacturer's instructions. Briefly, cells were lysed in buffer A and placed in a filter cartridge. After centrifugation at 14,000 rpm for 30 s, pellets were resuspended and centrifuged at 3,000 rpm for 1 min. The supernatant was collected and centrifuged again at 14,000 rpm for 30 min. The pellet was collected as total membrane fraction including organelles and plasma membranes. The total membrane protein fraction was resuspended in buffer B and centrifuged at 10,000 rpm for 5 min. The supernatant was then mixed with 1.6 mL cold PBS and centrifuged again at 14,000 rpm for 30 min. The pellet was collected as plasma membrane (PM) protein fraction for immunoblotting.

### Statistical analysis

Data were expressed as mean ± SD. All data were independently repeated for at least 3 times. Statistical analysis was carried out using a two-tailed unpaired t-test with GraphPad Prism software. p < 0.05 was considered as statistically significant.

## Supplementary Material

Supplementary materials and methods, figures and table.
