# Supplementary material for: Oncogenic β-catenin stimulation of cofilin 1-mediated macropinocytosis is druggable for cancer
Source: Theranostics. 2025 Mar 17;15(9):4176–87. doi: 10.7150/thno.104283 (PMC11980659; doi:10.7150/thno.104283)
Supplement: Supplementary file 1 — Supplementary materials and methods, figures and table. [file thnov15p4176s1.pdf]

# Supplementary Material for

# Oncogenic β-catenin stimulation of cofilin 1-mediated macropinocytosis is druggable for cancer

Baohui Zhang[1,2*], Xiaochen Gai[2*], Bufu Tang[3*], Linlin Chen[4], Yuting Wu[2], Weiwei Deng[2], Jiate Liu[5] , Lanting Hao[6], Fangming Liu[2#], Hongbing Zhang[1,2,7#].

[1] Department of Physiology, School of Life Science, China Medical University, Shenyang, Liaoning, China.

[2] Department of Physiology, State Key Laboratory of Common Mechanism Research for Major Diseases, Haihe Laboratory of Cell Ecosystem, Institute of Basic Medical Sciences and School of Basic Medicine, Chinese Academy of Medical Sciences and Peking Union Medical College, Beijing, China.

[3] Department of Radiation Oncology, Zhongshan Hospital, Fudan University, Shanghai, China.

[4] School of Life Sciences, Westlake University, Hangzhou, Zhejiang, China.

[5] Department of Neurosurgery, Shengjing Hospital of China Medical University, Shenyang, Liaoning, China.

[6] Department of Surgical Oncology and General Surgery, The First Hospital of China Medical University, Shenyang, Liaoning, China.

[7] Hubei Provincial Key Laboratory of Tumor Microenvironment and Immunotherapy, College of Basic Medical Sciences, China Three Gorges University, Yichang, Hubei, China

[*] These authors contributed equally.

[#] **Correspondence:** Hongbing Zhang, hbzhang@ibms.pumc.edu.cn.

Fangming Liu, lfmpumc@163.com.

## Supplementary Materials and Methods

### Reagents and drugs

OSI-027 (S2624, Selleck), MOMIPP (T33467, TargetMol), BafA1 (T6740, TargetMol), Pri-724 (S8968, Selleck), Rapamycin (T1537, TargetMol) were dissolved in dimethyl sulfoxide (DMSO). 4',6-diamidino-2-phenylindole (DAPI) was purchased from Abcam (ab104139). FITC-dextran was obtained from Sigma-Aldrich (#53471). MitoTracker (C1048) and LysoTracker (C1046) were purchased from Beyotime.

### Cell proliferation assay

Cells were seeded into 96-well plates with 3000 MEFs or 5000 hepatic cell lines per well each. Cells were incubated with drugs or DMSO for 48 h. Then the absorbance at 450 nm was measured using a microplate reader after incubating cells in 90 μL of medium containing 10 μL CCK-8 for 2 h. The inhibition rate was normalized against DMSO treatment. Each assay was repeated at least three times.

### Western blotting

Cells from various treatment groups were washed with PBS and then cell lysates were prepared using SDS loading buffer with Protease and Phosphatase Inhibitor Cocktail (EDTA-Free) (#P002, New cell & molecular, China). The BCA protein assay (Beyotime Institute of Biotechnology, China) was used to measure protein concentration. Cell lysates were separated by SDS-PAGE gel and transferred to nitrocellulose filter (NC) membranes. The following primary antibodies were used: β-catenin (#9587, Cell Signaling Technology, USA), Cyclin D1 (#A19038, Abclonal), LGR5 (#A12327, Abclonal), DYKDDDDK-tag (MA1-91878, Invitrogen), CFL1 (#A1704, Abclonal), phosphor-CFL1-S3 (#AP0178, Abclonal), Mouse anti HA-tag (#AE008, Abclonal), TESK1 (GTX55987, GeneTex), Na,K-ATPase (#3010, Cell Signaling Technology), P70S6K (#2708S, Cell Signaling Technology), phosphor-P70S6K (#9505S, Cell Signaling Technology), AKT1 (#2967S, Cell Signaling Technology), phosphor-AKT1-Ser473 (#4060S, Cell Signaling Technology), S6 (#2217S, Cell Signaling Technology), phosphor-S6 (#2211L, Cell Signaling Technology), 4EBP1 (#9644S, Cell Signaling Technology), phosphor-4EBP1-T37/46 (#2855S, Cell Signaling Technology), β-Actin (C1620,

Santa Cruz Biotechnology, USA). The secondary antibodies were IRDye 680RD goat anti-rabbit (#68071, LI-COR Biosciences, USA) and IRDye 800CW goat anti-mouse (#32210, LI-COR Biosciences). Blot signals were detected using LI-COR Odyssey Infrared Scanner.

**RNA extraction and real-time quantitative PCR**

Total RNA from the samples was extracted using TRIzol reagent (Invitrogen, USA) following the manufacturer's protocol. 2 μg RNA was reverse transcribed into complementary DNA (cDNA) using Hifair® Ⅱ 1st Strand cDNA Synthesis Kit (gDNA digester plus) (#11121ES60, Yeasen). The synthesized cDNA was amplified with quantitative real-time PCR using SYBR High-Sensitivity qPCR SuperMix (#abs60086, Absin Biosciences, China) on CFX Connect Real-Time PCR System (Bio-Rad). Relative mRNA expression was normalized to β-actin expression. Specific qPCR primer sequences are listed as follows:

Mouse *Ccnd1*-F: 5'-GCGTACCCTGACACCAATCTC-3'
Mouse *Ccnd1*-R: 5'-CTCCTCTTCGCACTTCTGCTC-3'
Mouse *Cfl1*-F: 5'-ATGACATGAAGGTTCGCAAGT
Mouse *Cfl1*-R: 5'-GACAAAAGTGGTGTAGGGGTC
Mouse *β-actin*-F: 5'-AGAGGGAAATCGTGCGTGAC-3'
Mouse *β-actin*-R: 5'-CAATAGTGATGACCTGGCCGT-3'
Human *CCND1*-F: 5'-GCTGCGAAGTGGAAACCATC-3'
Human *CCND1*-R: 5'-CCTCCTTCTGCACACATTTGAA-3'
Human *CFL1*-F: 5'-GGTGCCCTCTCCTTTTCGTT-3'
Human *CFL1*-R: 5'-GGCATAGCGGCAGTCCTTAT-3'
Human *β-ACTIN*-F: 5'-CCTGGCACCCAGCACAAT-3'
Human *β-ACTIN*-R: 5'-GGGCCGGACTCGTCATAC-3'

**Chromatin immunoprecipitation (ChIP)**

ChIP was performed in $\beta\text{-}catenin^{\Delta(ex3)/+}$ MEFs according to the instruction manual of the SimpleChIP Plus Enzymatic Chromatin IP Kit (Magnetic Beads) (#9005, Cell Signaling Technology). Cells seeded on 15 cm plates were crosslinked with 1% formaldehyde. The chromatin extracted was digested by micrococcal nuclease and

sonicated to an average size of 300-500 bp. Then the chromatin lysates were immunoprecipitated with 20 μL anti-β-catenin, 10 μL anti-Histone H3 or 2 μL anti-IgG antibody. Eluted DNA was quantified by qPCR using specific primers as follows:

Site1-F: 5'-GGGATGTCTCAAGCTGAGGG-3'

Site1-R: 5'-ACTGAGAAGAGGGTCTGCGA-3'

Site2-F: 5'-AAATGTGCCAAGTCGGGGAT-3'

Site2-R: 5'-GCCCACATTCGAACTCTCCA-3'

Site3-F: 5'-TACTCTGCGCCTCAAGCTCT-3'

Site3-R: 5'-CCGACGGGAAGTGAAGTCC-3'

Site4-F: 5'-ACAACGCCAAAGCTCTCAAAC-3'

Site4-R: 5'-GCTTGGGATCCTCTCTCACAG-3'

Site5-F: 5'-ACCCTTCCCCCATGAATTACG-3'

Site5-R: 5'-GTTTGAGAGCTTTGGCGTTGT-3'

**RNA interference**

Cells seeded on 6-well plates were transfected with siRNA oligonucleotides against β-catenin, CFL1 genes and non-specific control (100 nM) using Lipofectamin RNAiMax transfection reagent (Invitrogen, USA) according to the manufacturer's instructions. Cell protein and RNA samples were obtained after 48 h of transfection. The sequences information was followed:

si-*β-catenin*-1: 5'-TTGTTATCAGAGGACTAAAT-3'

si-*β-catenin*-2: 5'-TCTAACCTCACTTGCAATAAT-3'

si-*Cfl1*-1: 5'-GCCGCTATGCACTCTATGATG-3'

si-*Cfl1*-2: 5'-GCACTCTATGATGCAACCTAT-3'

**Lentiviral Vector construction and transfection**

lentiviral vectors encoding shRNA targeting cofilin-1 and a non-targeting control were procured from GeneChem (Shanghai, China). These vectors were engineered to express the enhanced green fluorescent protein (eGFP) as a reporter. β-catenin overexpressing and β-catenin × Flag-tag plasmids were constructed using lentiviruses

purchased from TSINGKE (Beijing, China). The cells were transfected with lentiviral suspension using transfection reagent according to the manufacturer's recommendations. After 72-96 h, transduction efficiency was confirmed by Western blot analysis

**Co-immunoprecipitation (Co-IP) assay**

Cells were lysed in IP lysis buffer and supernatant lysate were mixed with Dynabeads primary antibody conjugates at 4°C overnight, which were prepared by binding 2–4 μg antibody to 50 μL of beads suspension. Antibodies used for IP were CFL1 antibody (sc-53934, Santa Cruz Biotechnology), β-catenin antibody (#9587, Cell Signaling Technology) or normal mouse IgG (#5127, Cell Signaling Technology). The co-immunoprecipitated proteins were washed five times with IP wash buffer and eluted in 2 × LDS-containing protein sample buffer. After heating at 95°C for 5 min, the eluted proteins were then subjected to SDS-PAGE and Western blotting.

**Immunofluorescent staining (IF)**

Cells were washed with PBS and fixed with 4% paraformaldehyde at room temperature for 30 min. 0.3% Triton X-100 in TBS was used to permeabilize cells at room temperature for 1 hour. Then cells were incubated with primary antibodies overnight at 4℃. On the second day, the fluorescein-conjugated secondary antibodies were incubated with samples for 1 hour at room temperature. The slides were mounted with DAPI after three times washes with PBS. Images were captured using a fluorescence microscope (Leica, Germany).

**Subcutaneous tumor in nude mice**

Our study examined male and female animals, and similar findings are reported for both sexes. Female Balb/c nude mice (4-6 weeks) were obtained from HFK Bio-Technology. *β-catenin*$^{\Delta(ex3)/+}$ MEFs were diluted at the density of $1\times10^6$ cells in 100 μL PBS, and were injected subcutaneously into the back of nude mice using a BD disposable sterile syringe. Tumor formation in nude mice was observed every 3 days and drug treatment was given when tumors grew to 4 to 5 mm in diameter. The mice were randomly divided into two groups and then treated by intraperitoneal injection with DMSO control and OSI-027 (15 mg/kg) with five days a week. Body weight and

tumor volume of mice were continuously recorded. Tumor volume (V) = length × width$^2$/2. When the tumor volume reached 1000 mm$^3$, mice were sacrificed through cervical dislocation and the subcutaneous tumor was removed, photographed.

**Establishment of hepatocellular carcinoma orthotopic model in C57BL/6 mice**

Hepa1-6 cells were diluted in PBS to the density of $1\times10^8$ cells /mL and placed on ice for later use. C57BL/6 mice aged 7-8 weeks were under anesthetic using air-anesthesia machine. Using sterile ophthalmic scissors, the left lobe of liver was exposed from 1 cm incision under the xiphoid. Tumor cell suspension (20 μL, ~$2\times10^6$ cells) was then gently and slowly injected into liver parenchyma. After 30 seconds of cotton pressure to stop bleeding, the abdomen was closed layer by layer using medical biological glue. Mice were placed in the incubator immediately after the operation and treated with intraperitoneal injection after the wound healed completely. Body weight was monitored twice a day. When the abdominal enlargement of mice was visible or the hard abdominal mass was touched, mice were sacrificed by cervical dislocation and the size, weight and number of nodules of liver tumors were recorded. All animal studies were performed following the protocols approved by the Animal Center of the Institute of Basic Medical Sciences, Chinese Academy of Medical Sciences and Peking Union Medical College (ethical code: ACUC-A02-2022-057).

**Supplementary Figure 1. OSI-027 inhibits β-catenin active cells without involving mTOR signaling.**

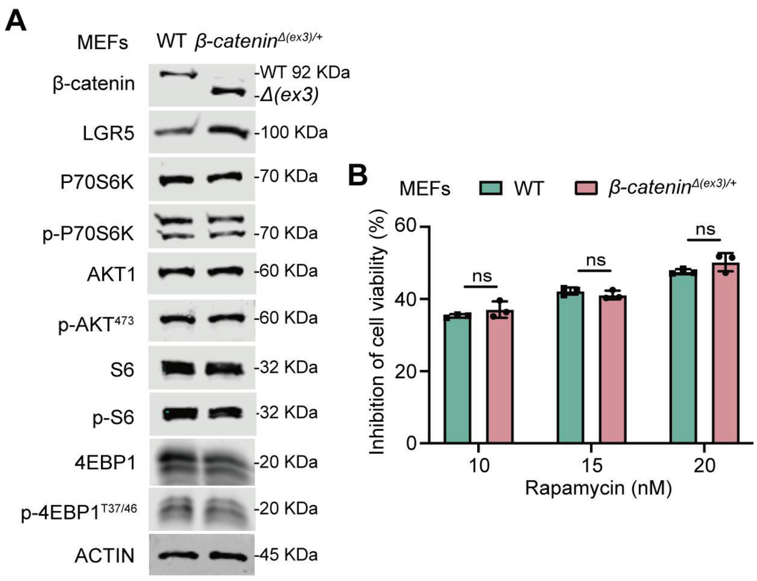


**(A)** Immunoblot analysis of β-catenin and mTOR signaling pathways in WT and *β-catenin*$^{\Delta(ex3)/+}$ MEFs. **(B)** Inhibition rate of rapamycin on WT and *β-catenin*$^{\Delta(ex3)/+}$ MEFs. Cells were treated with rapamycin at various concentrations for 48 h. n = 3. Data were shown as mean ± SD and analysis was performed using t test. *n.s.* no significance.

**Supplementary Figure 2. OSI-027-induced vacuoles are correlated with macropinocytosis.**

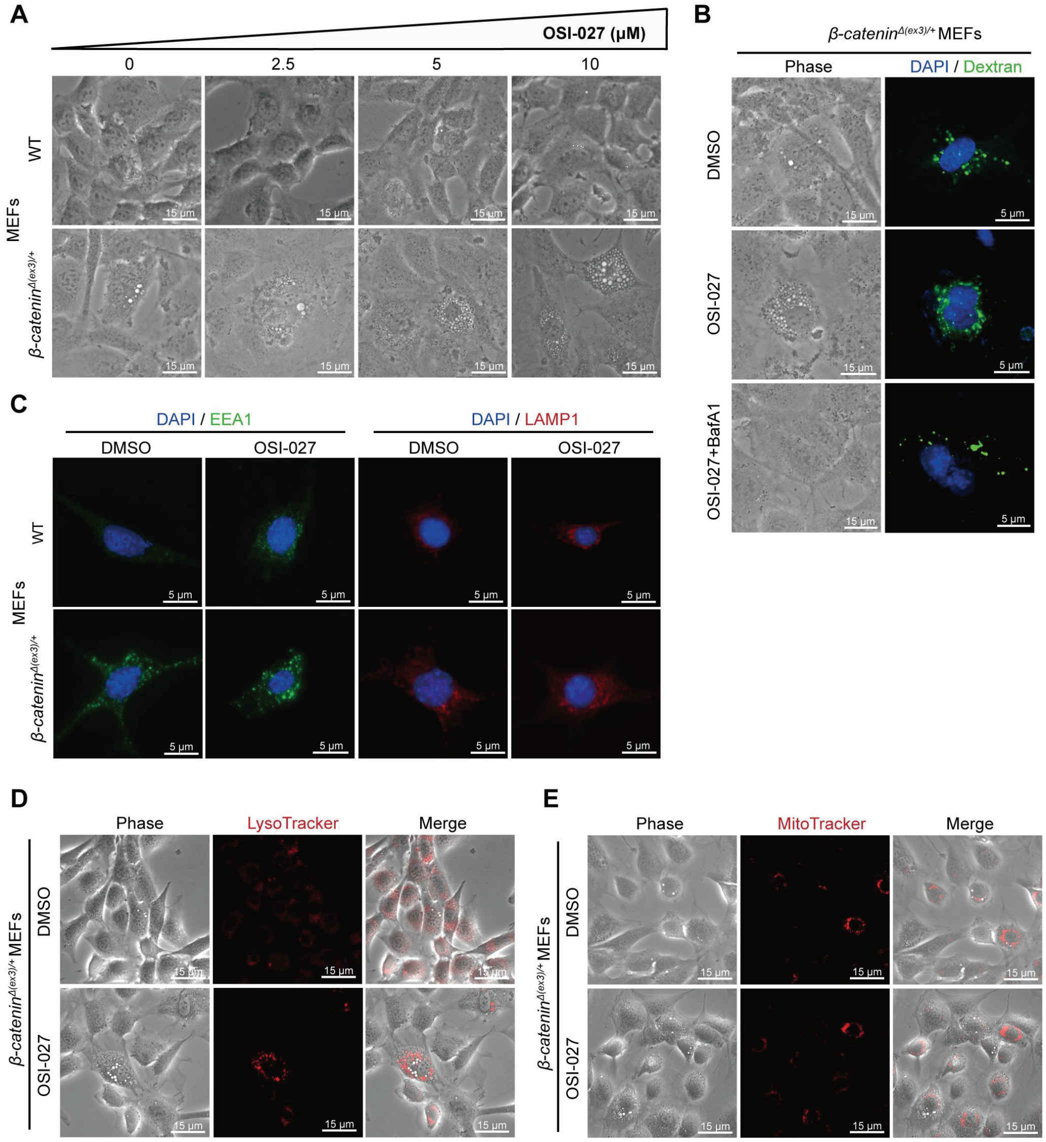


**(A)** Phase-contrast images of MEFs treated with OSI-027 (48 h) in a dose-dependent manner (2.5, 5, 10 μM). Scale bars, 15 μm (20 × image). **(B)** Phase-contrast and fluorescent images of *β-catenin*$^{\Delta(ex3)/+}$ MEFs incubated for 30 min with 1 mg/mL FITC-dextran (green). *β-catenin*$^{\Delta(ex3)/+}$ MEFs were pretreated with BafA1 (100 nM) prior to addition of DMSO or OSI-027 (2.5 μM). DAPI (blue). **(C)** Fluorescence immunostaining for EEA1 (green) and LAMP1 (red) in MEFs treated with DMSO or OSI-027 (2.5 μM, 48 h). DAPI (blue). Scale bars, 5 μm (40 × image). **(D-E)** Localization of subcellular markers in *β-catenin*$^{\Delta(ex3)/+}$ MEFs treated with DMSO or

OSI-027 (2.5 μM, 48 h). *$\beta$-catenin*$^{\Delta(ex3)/+}$ MEFs were incubated with 50 nM LysoTracker Red **(D)** and 75 nM MitoTracker Red **(E)** for 30 min at 37°. Phase-contrast image (left), fluorescent images (middle), merge images (right). Scale bars, 15 μm (20 × image).

**Supplementary Figure 3. MOMIPP induces cell death with the characteristics of methuosis in β-catenin active cells.**

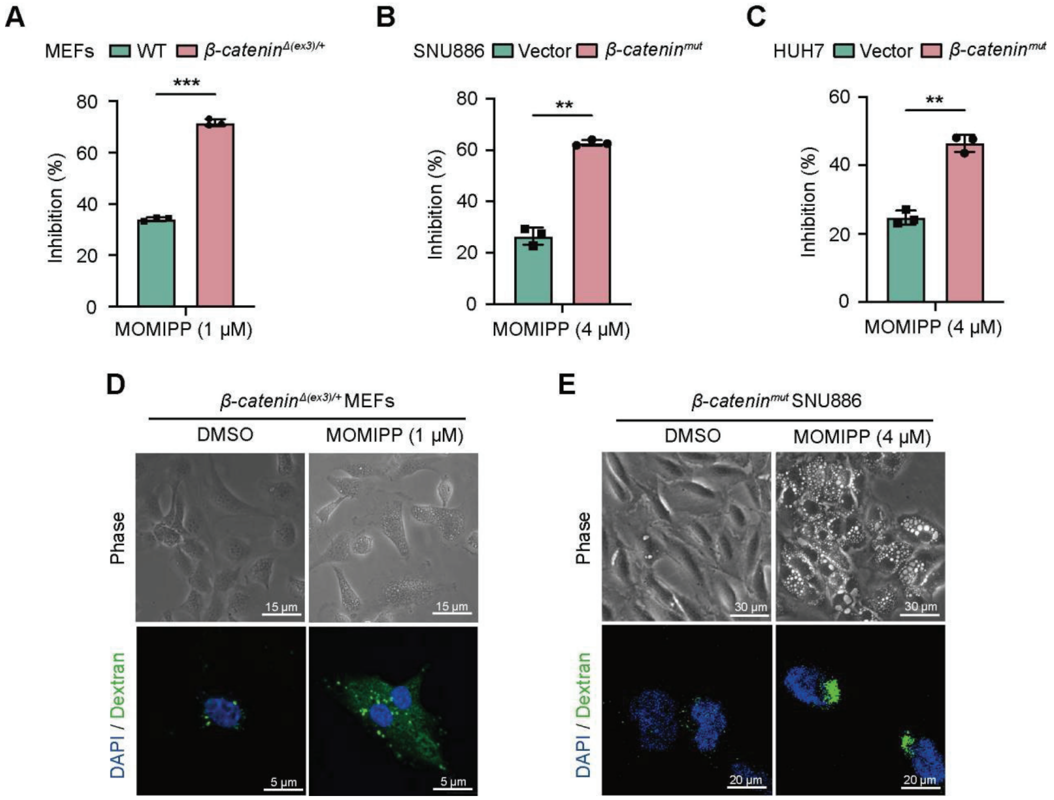


**(A-C)** Inhibition rate. MEFs **(A),** SNU886 **(B)** and HUH7 **(C)** cells transfected with vector or *β-catenin*$^{mut}$ plasmid were treated with DMSO or MOMIPP for 48 h. n = 3. **(D-E)** Phase-contrast and FITC-dextran uptake of *β-catenin*$^{Δ(ex3)/+}$ MEFs **(D)** and *β-catenin*$^{mut}$ SNU886 cells **(E)**. Cells were treated with DMSO or MOMIPP for 48 h, followed by incubation with FITC-dextran (1 mg/mL) for 30 min. FITC-dextran (green), DAPI (blue). Data were shown as mean ± SD and analysis was performed using *t test*. $**p < 0.01$, $***p < 0.001$.

**Supplementary Figure 4. β-catenin transcriptionally activates CFL1.**

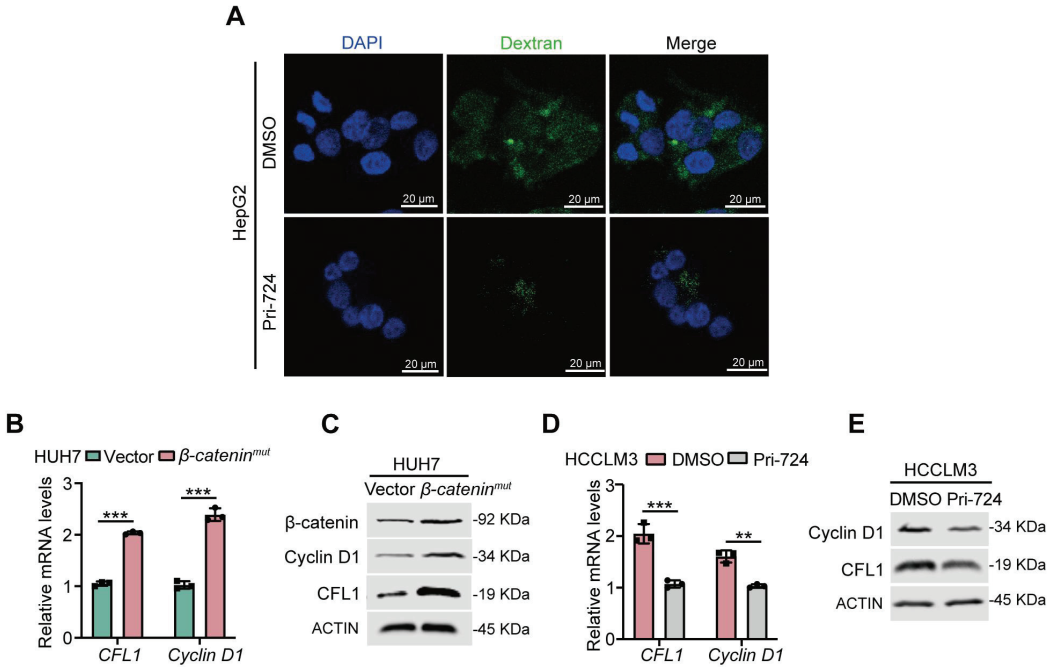


**(A)** FITC-dextran uptake of HepG2 cells. Cells were treated with DMSO or Pri-724 (20 μM) for 24 h and then incubated with FITC-dextran (1 mg/mL) for 30 min. FITC-dextran (green), DAPI (blue). Scale bars, 20 μm (40 × image). **(B-C)** *CFL1* mRNA levels **(B)** and protein levels ***(C)*** in HUH7 transfected with vector or *β-catenin*$^{mut}$ plasmid. **(D-E)** *CFL1* mRNA levels **(D)** and protein levels **(E)** in HCCLM3 treated with DMSO or Pri-724 (20 μM) for 48 h. Data were shown as mean ± SD and analysis was performed using *t test*. ***p* < 0.01, ****p* < 0.001.

**Supplementary Figure 5. β-catenin activation reduces CFL1 phosphorylation.**

A

| Sample Name | Position | Mut/WT Ratio | Regulated Type | Amino acid | Gene name | Localization probability | Modified sequence |
|---|---|---|---|---|---|---|---|
| *β-catenin*$^{\Delta(ex3)/+}$/WT | 3 | 0.108 | Down | S | Cfl1 | 0.999994 | AS(1)GVAVSDGVIK |

LC/MS analysis of the phosphorylation sites of CFL1 in MEFs.

**Supplementary Figure 6. CFL1 and phospho-CFL1 levels in whole cell or cell membrane of β-catenin active hepatic cells.**

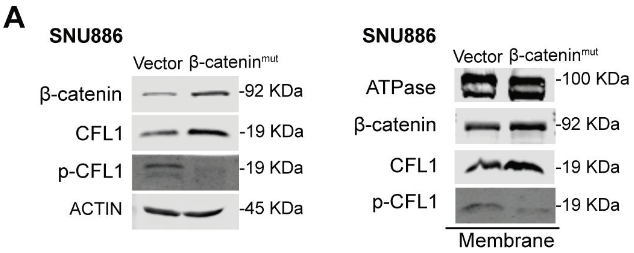


**(A)** CFL1 and phospho-CFL1 levels in whole cells or cell membranes were examined by immunoblotting. HUH7 cells were transfected with vector or *β-catenin*$^{mut}$ plasmid. (Total, left; membrane, right).

**Supplementary Figure 7. CFL1 is required for active β-catenin-stimulated macropinocytosis.**

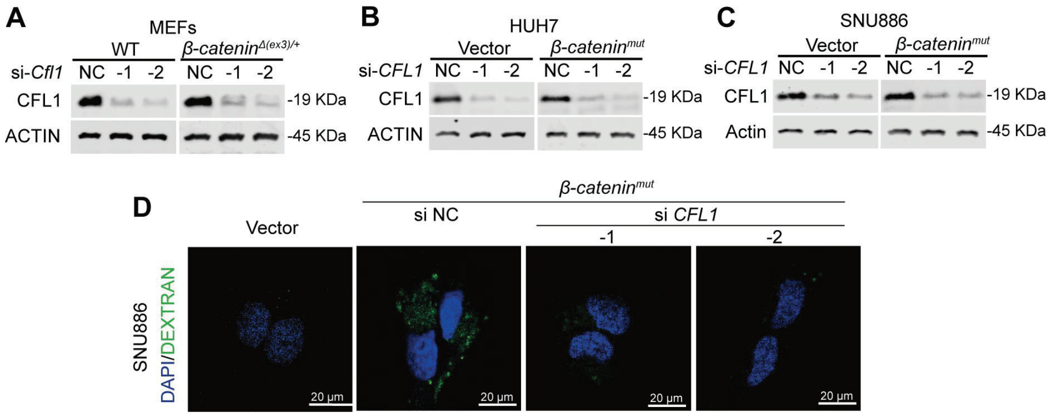


**(A-C)** CFL1 protein level was measured by western blot analysis after cells were transfected with control or *Cfl1* siRNA. **(A)** WT and *β-catenin*$^{\Delta(ex3)/+}$ MEFs. **(B)** Vector or *β-catenin*$^{mut}$ plasmid-transfected HUH7 cells. **(C)** Vector or *β-catenin*$^{mut}$ plasmid-transfected SNU886 cells. **(D)** FITC-dextran uptake. *β-catenin*$^{mut}$ plasmid-transfected SNU886 cells were treated with control or *CFL1* siRNA. FITC-dextran (green), DAPI (blue). Scale bars, 20 μm (40 × image).

**Supplementary Figure 8. CFL1 is required for HCC development.**

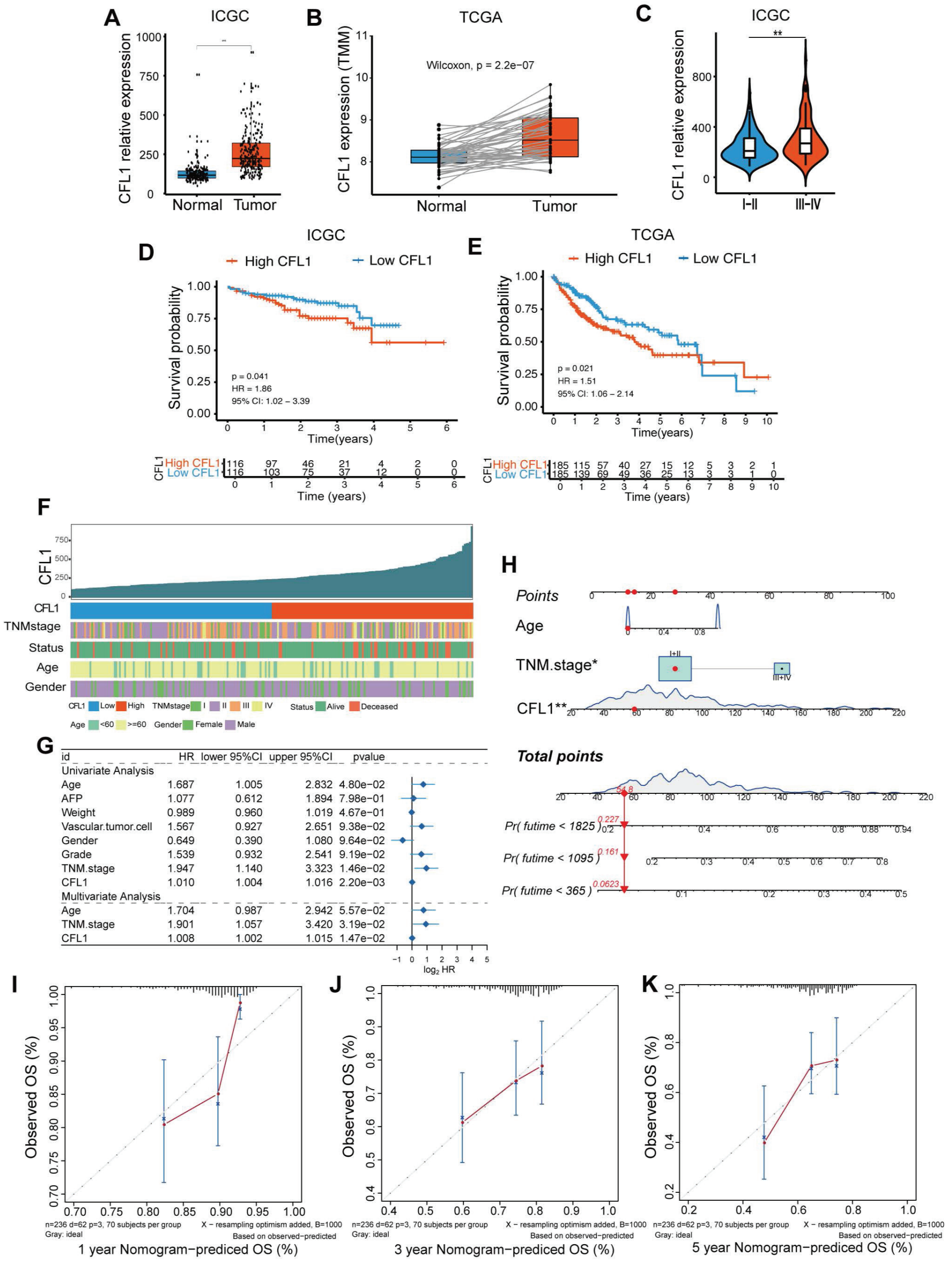


**(A-C)** Correlation of CFL1 level with tumors and clinical stages of HCC patients from ICGC and TCGA databases. **(D-F)** Correlation of CFL1 level with the overall survival (OS) **(D, E)** and clinical characteristics **(F)** of HCC patients. **(G)** Forest plot for multivariate Cox regression analysis. **(H)** Nomogram combining clinicopathological variables and CFL1 score predicts 1-, 3-, and 5-year OS of HCC patients. **(I-K)** The calibration plot for the internal validation of the nomogram.

**Supplementary Figure 9. OSI-027 inhibits tumor growth in nude mice.**

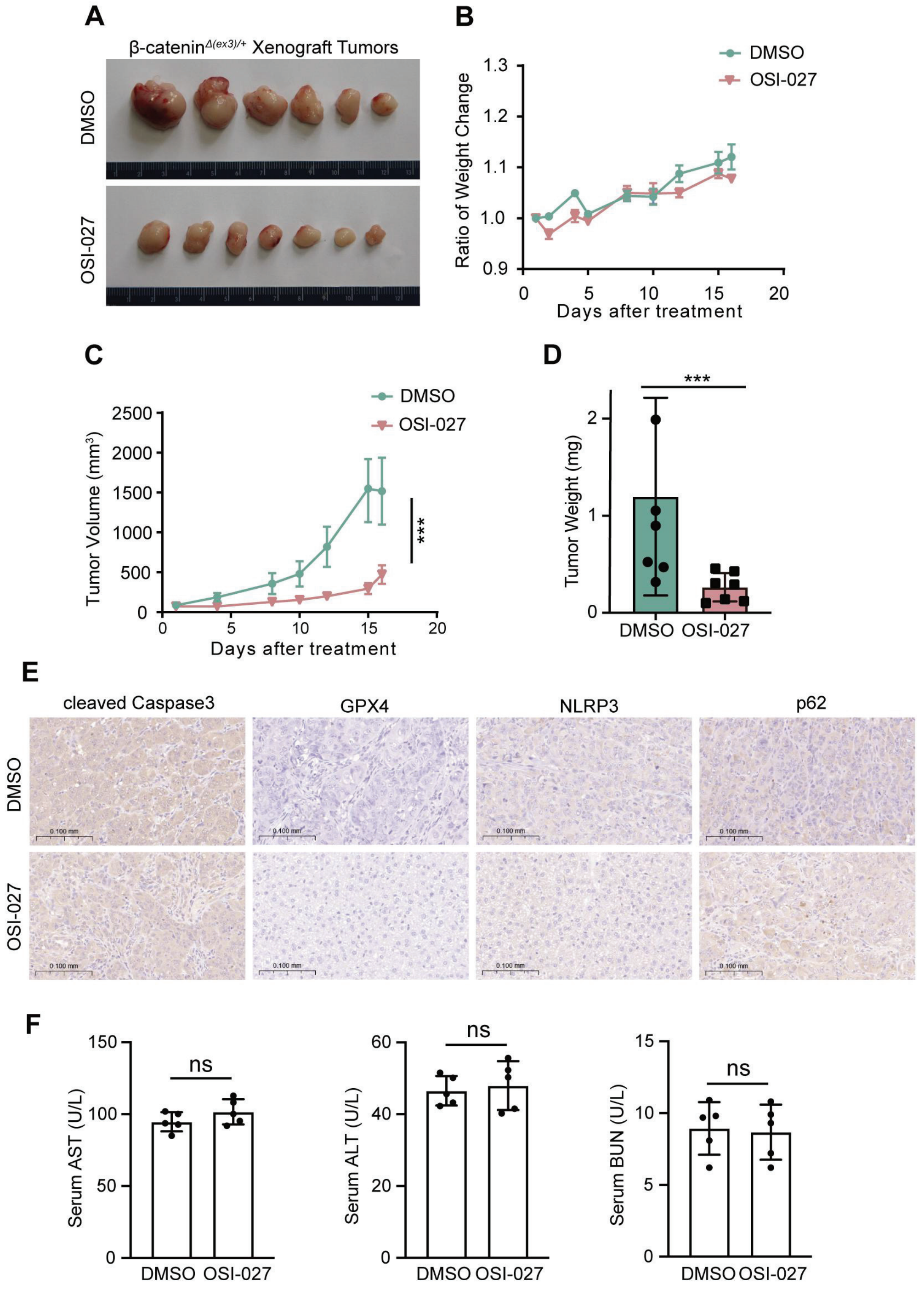


**(A-F)** Nude mice subcutaneously inoculated with *β-catenin*$^{\Delta(ex3)/+}$ MEFs were administered intraperitoneally with PBS (n=6) or OSI-027 (n=7, 15 mg/kg, 5 times one week). **(A)** Tumor images, **(B)** Ratio of body weight change, **(C)** Tumor growth was plotted as the mean change in tumor volume, **(D)** Tumor weight. **(E)**

Representative IHC staining of cell death markers in tumors tissues of nude mice with *β-catenin*$^{Δ(ex3)/+}$ MEFs. cleaved-caspase-3 was related to apoptosis; GPX4 was related to ferroptosis; NLRP3 was related to pyroptosis; p62 was related to autophagy. **(F)** Levels of AST, ALT, and BUN in each treatment group of tumor-bearing nude mice. Data were shown as mean ± SD and analysis was performed using *t test*. $^{**}p < 0.01$, $^{***}p < 0.001$.

**Table S1 Mutations of cell lines**

| Cell Line | Species | Disease | Mutation | | | |
|---|---|---|---|---|---|---|
| | | | ***CTNNB1*** | ***APC*** | ***AXIN2*** | ***AXIN1*** |
| Huh7 | *Homo sapiens* | HCC | no | no | no | no |
| SNU886 | *Homo sapiens* | HCC | no | no | no | no |
| Hepa 1-6 | *Mus musculus* | Hepatoma | YES | unknown | unknown | unknown |
| HepG2 | *Homo sapiens* | Hepatoblastoma | YES | no | no | no |
| HCCLM3 | *Homo sapiens* | HCC with high metastatic potential | YES | unknown | unknown | unknown |
